# RIS-1/psoriasin expression in epithelial skin cells indicates their selective role in innate immunity and in inflammatory skin diseases including acne

**DOI:** 10.1080/19381980.2017.1338993

**Published:** 2017-10-04

**Authors:** Christos C. Zouboulis, Claudia Beutler, Hans F. Merk, Jens M. Baron

**Affiliations:** a Departments of Dermatology, Venereology, Allergology and Immunology, Dessau Medical Center, Brandenburg Merdical School Theodore Fontane, Dessau, Germany; b Julius Wolff Institute for Biomechanics and Musculosceletal Regeneration, Charité Universitaesmedizin Berlin, Berlin, Germany; c Department of Dermatology and Allergology, RWTH Aachen University, Aachen, Germany

**Keywords:** RIS-1, psoriasin, acne, keratinocytes, sebocytes, bacteria

## Abstract

Objective: RIS-1/psoriasin/S100A7 is an epithelial antimicrobial peptide, whose expression is upregulated in inflammatory skin diseases and is induced by retinoids. Its molecular expression was investigated in skin cell cultures and in skin specimens to better understand its role in inflammatory procedures of the pilosebaceous unit. Methods: rtPCR and northern blotting of RIS-1/psoriasin and the retinoid-metabolizing genes CYP26AI and CRABP-II were performed in cells cultures (keratinocytes, sebocytes, fibroblasts, endothelial cells, melanocytes, lymphocytes and prostate cells; native and treated with retinoids) and in situ hybridization in normal and inflamed skin (acne, psoriasis). Results: a) RIS-1/psoriasin is expressed in keratinocytes and fibroblasts *in vitro* and in keratinocytes of the stratum granulosum *in vivo*. Retinoids *in vitro* and inflammatory conditions *in vivo* increase the levels of RIS-1/psoriasin in keratinocytes (both), sebocytes (inflammation only) and fibroblasts (retinoids). Sebocytes and fibroblasts are the metabolically most active skin cells, since they can upregulate the expression of CRABP-II and CYP26AI, genes responsible for retinoid metabolism. Inflammation modifies the compartmentation of RIS-1/psoriasin in sebaceous glands and the follicular root sheaths. Conclusion: The present data indicate that anti-inflammatory treatment targeting the epithelial compartments of the skin, including such with antibacterial peptides, may be promising for inflammatory skin diseases.

## Introduction

Psoriasin (S100A7) belongs to the family of S100 proteins, which constitute a family of low molecular weight calcium binding proteins of the human epithelia, located on chromosome 1q21 in a gene cluster known as epidermal differentiation complex.^1-3^ It was first discovered overexpressed in psoriasis,^4^ but was later detected to be upregulated in several inflammatory skin diseases,^5^ and its protein has been found to be a selective chemokine for CD4+ T lymphocytes and neutrophils.^6^ Moreover, psoriasin has initially been characterized as a retinoid-inducible skin-specific gene (RIS-1).^7,8^

Nowadays, RIS-1/psoriasin/S100A7 is also recognized as an antimicrobial peptide produced by keratinocytes against bacteria.^9,10^ In favor of this, *in vivo* treatment of human skin with antibodies to psoriasin inhibited its *E. coli*-killing properties.^11^

On the other hand, increasing *in vitro* and* in vivo* evidence demonstrates that commensal bacteria, such as Propionibacterium acnes and Staphylococcus epidermidis, continuously induce a certain level of innate immunity in the skin to keep the defense against pathogens awaken.^12-14^ This skin function is mostly expressed in acne, the most common disease of the pilosebaceous unit.^15,16^

Therefore, we decided to investigate the expression of RIS-1/psoriasin in skin cell cultures and in the skin at the molecular level to better understand its role in inflammatory skin conditions, especially those of the pilosebaceous unit.

## Results

### Expression RIS-1/psoriasin in human skin cells in vitro

**Figure 1.**
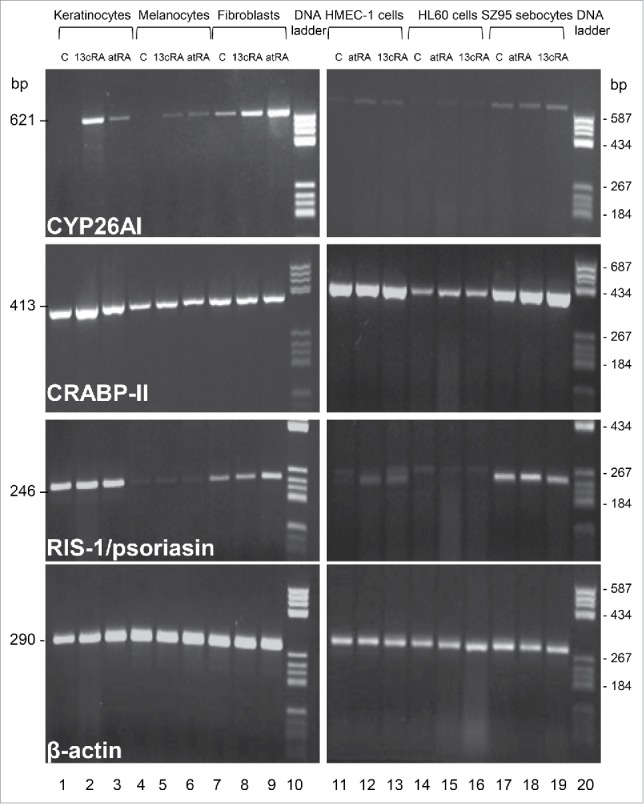
rtPCR detection of CYP26AI, CRABP-II and RIS-1/psoriasin mRNA expression. Lanes 1–3, primary human keratinocytes; lanes 4–6, primary human melanocytes; lanes 7–9, primary human fibroblasts, lane 10, DNA mass ladder; lanes 11–13, human immortalized vascular endothelial cell line HMEC-1; lanes 14–16, human promyelocytic leukemia cell line HL-60, lanes 17–19, human immortalized SZ95 sebocytes; lane 20, DNA mass ladder. DMSO control (C, lanes 1, 4, 7, 11, 14, 17), 13*cis*-retinoic acid in DMSO (13cRA, 10^−7^ M; lanes 2, 5, 8, 13, 16, 19), all-*tran*s retinoic acid in DMSO (atRA, 10^−7^ M; lanes 3, 6, 9, 12, 15, 18).

The expression of RIS-1/psoriasin in human skin cells *in vitro* at the mRNA level was initially screened by reverse transcription polymerase chain reaction (rtPCR). RIS-1/psoriasin is expressed in primary keratinocytes, SZ95 sebocytes and primary fibroblasts, but not in primary melanocytes, the immortalized vascular endothelial cell line HMEC-1^17^ and the human promyelocytic leukemia cell line (HL-60) (Fig. 1). Retinoids seemed to upregulate RIS-1/psoriasin mRNA expression in fibroblasts and HMEC-1 endothelial cells. In contrast, cellular retinoic acid-binding protein 2 (CRABP-II), a retinoid-inducible gene, facilitating retinoid transport and metabolism,^8^ was expressed in all cell types tested. Cytochrome P45026A1 (CYP26AI), a gene indicating active retinoid metabolism,^18^ was expressed in SZ95 sebocytes and fibroblasts and was upregulated by retinoids in all cell types tested except of the HL60 cells.

**Figure 2.**
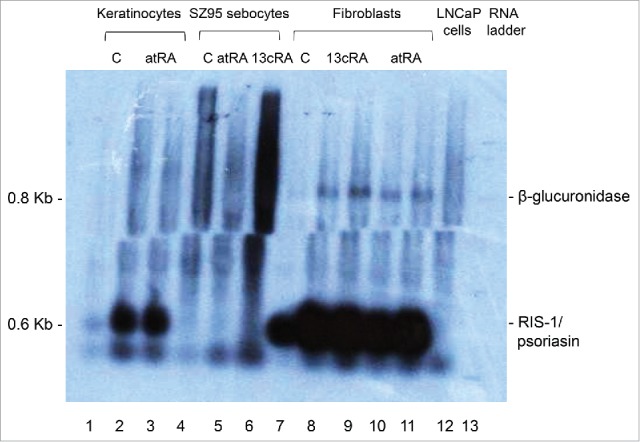
Northern blot detection of RIS-1/psoriasin mRNA levels. Lanes 1–3, primary human keratinocytes; lanes 4–6, human immortalized SZ95 sebocytes; lanes 7–11, primary human fibroblasts, lane 12, human prostatic carcinoma LNCaP cell line; lane 13, RNA mass ladder. DMSO control (C; lanes 1, 4, 7, 13), all-*trans* retinoic acid in DMSO (atRA, 10^−7^ M; lanes 2, 3, 5, 6, 10, 11), 13*cis*-retinoic acid in DMSO (13cRA, 10^−7^ M; lanes 8, 9).

Northern blot studies confirmed that RIS-1/psoriasin is mainly expressed at the mRNA level in fibroblasts and at a lower level in keratinocytes (Fig. 2). In all other cell types tested, including the human prostatic carcinoma LNCaP cell line, RIS-1/psoriasin mRNA expression was very low on not detectable. Moreover, retinoids induced RIS-1/psoriasin mRNA expression in fibroblasts and keratinocytes.

### Expression RIS-1/psoriasin in human skin cells in vivo

**Figure 3.**
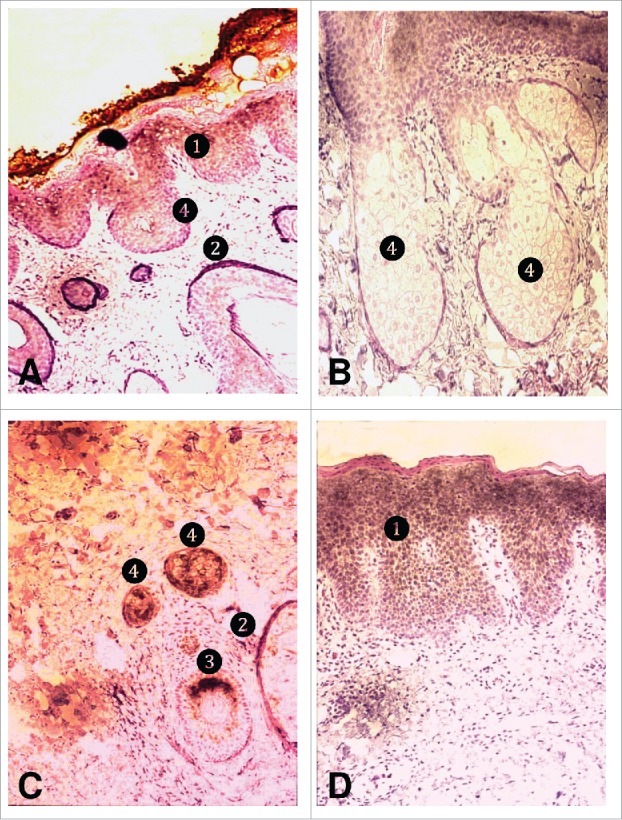
Detection of cellular compartmentation of RIS-1/psoriasin mRNA expression by in situ hybridization. A) Normal skin (40x), B) normal sebaceous glands (100x), C) acne skin (100x), d) psoriatic skin (40x). (1) Stratum granulosum; (2), follicular outer root sheath; (3) follicular inner root sheath; (4) sebaceous gland.

A strong, focal RIS-1/psoriasin mRNA expression was detected by in situ hybridization at the epidermal statum granulosum and the outer root sheath of the hair follicles (Fig. 3). RIS-1/psoriasin mRNA could also be visualized in fibroblasts and single basal sebocytes.

In acne skin, a polarized, strong RIS-1/psoriasin mRNA expression was also detected in the inner root sheath of the hair follicles near the area of the ductus seboglandularis and in differentiating sebocytes. At last, in psoriatic skin, a strong RIS-1/psoriasin mRNA expression was extended in the widened stratum granulosum.

## Discussion

According to the major findings of the current work a) RIS-1/psoriasin is not only expressed in cultured human keratinocytes^3,4,7^ but also in fibroblasts, whereas the mRNA expression levels are higher than those of keratinocytes *in vitro*. However, *in vivo* keratinocytes of the stratum granulosum express much higher RIS-1/psoriasin mRNA levels^3,4,7^ than fibroblasts, indicating, that the genuine expression of RIS-1/psoriasin in fibroblasts is inhibited *in vivo*.

b) RIS-1/psoriasin expression is recognized in certain skin cells,^3^ since neither other human skin cells, such as melanocytes and endothelial cells, nor professional inflammatory cells and epithelial cells of non-cutaneous origin, such as prostate cells, express RIS-1/psoriasin.
c) Retinoids *in vitro* and inflammatory conditions *in vivo* (acne, psoriasis) increase the levels of RIS-1/psoriasin mRNA expression in keratinocytes (both),^47^ and in sebocytes (inflammation only), which are cells been involved in the induction of innate skin immunity. Interestingly, RIS-1/psoriasin has been detected in human vernix caseosa and amniotic fluid, an implication for newborn innate defense.^19^ Indeed, it is the vernix caseosa, produced by the hypertrophic embryonal sebaceous glands which has been shown to protect the embryonic skin in utero at the third trimenon of pregancy.^20^
d) Sebocytes and fibroblasts are apparently the metabolically most active skin cells, since they express detectable mRNA levels and can upregulate the expression of genes responsible for retinoid metabolism, such as CRABP-II and CYP26AI, confirming previous results.^21,22^
e) While epidermal keratinocytes of the stratum granulosum are expressing RIS-1/psoriasin in both homeostatic and inflammatory conditions, this function is attributed in different compartments of the pilosebaceous unit. Follicular keratinocytes of the outer root sheath and undifferentiated sebocytes express RIS-1/psoriasin in homeostasis but polarized follicular keratinocytes of the inner root sheath near the ductus seboglandularis and differentiating sebocytes overtake at an inflammation status. Even if the latter confirms already published data,^3^ the switch of responsible cells in a skin compartment to fulfill anti-inflammatory activities represents new knowledge.

Our data indicate that anti-inflammatory treatment targeting the epithelial compartments of the skin, including such with antibacterial peptides, may be promising for inflammatory skin diseases.^23^ Indeed, initial clinical studies with antibacterial peptide derivatives already have been demonstrated to be effective in the treatment of acne.^24^

## Materials and methods

### Cell cultures

Primary human keratinocytes and the immortalized human SZ95 sebaceous gland cell line (Zouboulis et al, 1999) were maintained in Dulbecco's modified Eagle's (DME)/Ham's F 12 medium (1:1; Biochrom, Berlin, Germany) with 2 mM N-acetyl-L-alanyl-L-glutamine, supplemented with 10% heat-inactivated fetal calf serum (FCS; Biochrom) and 50 µg/ml gentamycin (Gibco-BRL, Karlsruhe, Germany) at 37°C in a humidified atmosphere containing 5% CO_2_ until reaching 70% confluence. The cells were then washed with phosphate-buffered saline without Ca^2+^ and Mg^2+^ (PBS; Biochrom), detached with trypsin/ethylendiamine tetraacetic acid 0.05%/0.02% (Gibco-BRL) and were subcultured. For all studies, keratinocyte subculture 4 and SZ95 sebocyte subculture 27 were used.

Before performing the experiments, the cells have been accustomed to diminished FCS concentrations and have been finally subcultured twice in serum-free DME/Ham's F 12 medium only supplemented with N-acetyl-L-alanyl-L-glutamine and antibiotics.

Primary human fibroblasts, primary human melanocytes, the human promyelocytic leukemia cell line (HL-60), the immortalized vascular endothelial cell line HMEC-1^17^ and the human prostatic carcinoma LNCaP cell line were used as controls.

### Treatment with retinoids

All-*trans* retinoic acid atRA (atRA) and 13-cis retinoic acid (13cRA) (Sigma Aldrich) were dissolved in dimethyl sulfoxide (DMSO). The final concentration of DMSO in medium was 0.2%. Cells cultured in medium supplemented with DMSO but without retinoids served as controls. The retinoid compounds and the cell cultures were handled under dimmed yellow light.

For rtPCR and RNA gel blot studies, medium (6 ml) supplemented with retinoids (10^−7^ M) or control medium was added to subconfluent cell cultures in 100 mm culture dishes (Falcon, Becton Dickinson) for 24 hours.

### RNA preparation

After aspiration of the medium 3.9 ml lysis buffer (RNeasy Kit, Qiagen) were added to the cells and total RNA was extracted according to the instruction manual of the RNeasy Kit. Specimens were stored at −80°C until used for the rtPCR experiments described below.

### rtPCR

Total RNA was quantitated spectrophotometrically and 10 ng were used for each reaction. rtPCR was performed in a hot-top Stratagene robocycler using the rtPCR Access Kit (Promega) according to the instruction manual. Specific primer pairs for RIS-1 (246 bp cDNA fragment), CYP26AI (621 bp) and CRABP II (413 bp) were used (Table 1). Controls using specific primers for β-actin (290 bp) confirmed comparable volumes of total RNA. All rtPCR products and a 100 bp DNA mass ladder (MBI Fermentas) were separated on 2% agarose gels and visualized by ultraviolet B light using ethidium bromide staining.

**Table 1.** Primers used for rtPCR analysis.

Gene	Sense primer location	Anti-sense primer location	PCR product (bp)
CYP26AI	AGG CAC TAA AGC AAT CTT CAA	CAT GGA AAT GGG TGA ATC TTG	621
CRABP-II	CAACTGGAAAATCA-TCCGA	CAACGTCATCCGC[C/T]GTCAT	413
RIS-1/psoriasin	TTAC-CTCGCCGACGTCTTTG	TCACTGGCTGCCC-CCGGAA	246
β-actin	ACCCACACTGTGCCCATCTA	CGGAACCGCTCATTGCC	290

### Northern analysis

Concentrations of total ENA were determined by absorbance at 260 nm and verified by nondenaturing agarose gel electrophoresis and ethidium bromide staining. 20 µg of total RNA was size-fractionated by electrophoresis in 1% formaldehyde-agarose gels containing 0.2 µg/ml ethidium bromide and transferred to derivatized nylon membranes (GeneScreenPlus, NEN). Complete transfer of RNA was documented by examining the gels under UV light. The blots were sequentially hybridized against RIS-1^7^ and β-glucuronidase cDNA probes labeled to a specific activity of 3 × 10^9^ cpm/µg DNA with ^32^P-dCTP (Amersham) by the random priming technique at a concentration of 4 × 10^5^ cpm/ml hybridization buffer. The prehybridization and hybridization procedures were performed according to standard protocols.

### *In situ* hybridization

Paraformaldehyde-fixed paraffin sections from acne, psoriatic and human scalp skin were hybridized with a digoxigenin-labeled specific probe using a commercial kit (Boehringer Mannheim). The sections were digested with 5 μg/ml proteinase K for 15 min and re-fixed in PBS containing 4% paraformaldehyde. After rinsing in PBS, these sections were treated with 0.2 M HCl for 10 min, 0.1 M triethanolamine–HCl (TEA, pH 8.0) for 1 min and 0.1 M TEA containing 0.25% acetic anhydride for 10 min. The sections were then dehydrated by rinsing in ethanol solutions (70%, 80%, 90% and 100%) and air-dried. Hybridization with the RIS-1 probe was performed at 50°C overnight in 10 mM Tris–HCl (pH 7.6) containing 50% formamide, 100 μg/ml tRNA, 1 X Denhardt's solution, 2.5% dextran sulfate, 0.6 M NaCl, 0.25% SDS and 1 mM EDTA. After treatment with 5 μg/ml RNase A at 37°C for 30 min, final washing was performed with 0.2 X SSC at 50°C for 20 min.

mRNA expression was visualized after reaction with an alkaline phosphatase-labeled anti-digoxigenin antibody (Boehringer Mannheim).

All experiments performed in this study follow the principles of the Declaration of Helsinki.
